# Antibacterial Silver Nanoparticle Containing Polydopamine Hydrogels That Enhance Re-Epithelization

**DOI:** 10.3390/gels10060363

**Published:** 2024-05-24

**Authors:** Naphtali A. O’Connor, Abdulhaq Syed, Ertan Kastrat, Hai-Ping Cheng

**Affiliations:** 1 Department of Chemistry, Lehman College of the City University of New York, Bronx, NY 10468, USA; 2 Ph.D. Program in Chemistry, The Graduate Center, City University of New York, New York, NY 10016, USA; 3 Ph.D. Program in Biochemistry, The Graduate Center, City University of New York, New York, NY 10016, USAhaiping.cheng@lehman.cuny.edu (H.-P.C.)

**Keywords:** polydopamine, ionic crosslinking, silver nanoparticle, antibacterial, wound healing

## Abstract

A polydopamine polyelectrolyte hydrogel was developed by ionic crosslinking dextran sulfate with a copolymer of polyethyleneimine and polydopamine. Gelation was promoted by the slow hydrolysis of glucono-δ-lactone. Within this hydrogel, silver nanoparticles were generated in situ, ranging from 25 nm to 200 nm in size. The antibacterial activity of the hydrogel was proportional to the quantity of silver nanoparticles produced, increasing as the nanoparticle count rose. The hydrogels demonstrated broad-spectrum antibacterial efficacy at concentrations up to 10^8^ cells/mL for *P. aeruginosa*, *K. pneumoniae*, *E. coli* and *S. aureus*, the four most prevalent bacterial pathogens in chronic septic wounds. In ex vivo studies on human skin, biocompatibility was enhanced by the presence of polydopamine. Dextran sulfate is a known irritant, but formulations with polydopamine showed improved cell viability and reduced levels of the inflammatory biomarkers IL-8 and IL-1α. Silver nanoparticles can inhibit cell migration, but an ex vivo human skin study showed significant re-epithelialization in wounds treated with hydrogels containing silver nanoparticles.

## 1. Introduction

Polydopamine (PDA), commonly referred to as a ‘mussel-inspired’ adhesive, is synthesized through the oxidative oligomerization of polyphenol dopamine [1,2]. PDA is known for its biocompatibility and antioxidant properties [2,3], and has been shown to promote cell adhesion and proliferation [4]. It has attracted interest in recent years [5,6,7] because it can be easily coated onto a wide range of substrates, providing them with biocompatibility [8] and functional groups that can be developed for synthesis [9]. Functional groups present in PDA that facilitate surface modifications include phenols, amines, and imines.

In our previous work, we successfully developed a hydrogel composed of dextran covalently crosslinked with a polyethyleneimine-polydopamine copolymer [10]. This hydrogel was created through a one-pot two-step process that employed epichlorohydrin as the crosslinker, which necessitated a high-pH environment [11]. Although the hydrogels exhibited antioxidant properties and promoted cell migration in wound healing assays, the need for extensive washing to eliminate the toxic crosslinker and the use of sodium hydroxide (NaOH) limited their practical application. To overcome these limitations and establish a more biocompatible and environmentally friendly approach, here we transitioned to an ionic crosslinking strategy [12,13]. Here, we present an approach for the development of biocompatible, antimicrobial PDA hydrogels achieved through ionic crosslinking. Ionic crosslinking is a form of physical crosslinking, where oppositely charged molecules or polymers interact to create a polyelectrolyte complex [14]. Ionic crosslinked hydrogels have applications in various fields, including injectable hydrogels [15], bioprinting [16], and soft electronics [17]. This method eliminates the need for chemical crosslinkers, such as the commonly used glutaraldehyde or epichlorohydrin, which are often incompatible with biological systems. In our approach, PDA forms a copolymer with polyethyleneimine (PEI) [18] and acquires a cationic charge upon exposure to a reduced pH environment. Ionic crosslinking occurs in the presence of negatively charged polysaccharide, dextran sulfate, resulting in the formation of a polyelectrolyte hydrogel. This strategy can be readily adapted for injectable hydrogel applications that incorporate PDA.

PDA exhibits reductive properties and has been shown to produce silver nanoparticles (AgNPs) on thin film surfaces [19]. In our study, we leveraged the reductive capacity of polydopamine to synthesize AgNPs within the polyelectrolyte hydrogels, endowing them with broad-spectrum antibacterial activity against both Gram-positive and Gram-negative bacteria. Notably, these antibacterial AgNP-infused gels showed remarkable effectiveness against *Pseudomonas aeruginosa*, *Klebsiella pneumoniae*, *Escherichia coli*, and *Staphylococcus aureus*—the four most prevalent bacterial pathogens in chronic and septic wounds [20]. Examination of inflammation biomarkers interleukins IL-8 and IL-1α, revealed reduced expression in the presence of PDA-containing hydrogels, underscoring the critical role of PDA in ensuring biocompatibility [21]. Furthermore, an ex vivo wound healing study indicated that the AgNP-containing hydrogels promoted re-epithelization, further emphasizing their therapeutic potential.

## 2. Results and Discussion

### 2.1. Synthesis and Structure

**Figure 1:**
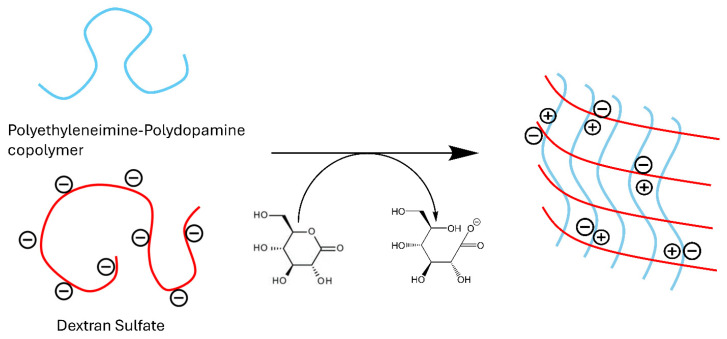
Ionic crosslinking of dextran sulfate with polyethyleneimine.

PEI is a polyamine that becomes a polycation in acidic media, which can be leveraged to form polyelectrolyte complexes with polyanions. To form polyelectrolyte complexes with PEI, our initial investigations explored the poly-anionic polymers alginate and chondroitin sulfate. However, dextran sulfate emerged as the most favorable candidate for complex formation. The process begins by thoroughly mixing neutral PEI with dextran sulfate. The solution is then acidified using glucono-δ-lactone, a neutral compound that hydrolyzes into gluconic acid in an aqueous solution (Figure 1). Glucono-δ-lactone is commonly used to slowly hydrolyze divalent cations for hydrogel crosslinking, but our use here represents its novel application to ionic crosslink polyanions with polyamines. The gradual release of protons plays a pivotal role in the gelation process, as the use of acids like HCl and acetic acid resulted in immediate precipitation. Optimal conditions were achieved with a mass ratio of 2:1 for PEI to dextran sulfate, as summarized in Table 1. Increasing the amount of dextran sulfate in the formulations resulted in softer gels, while an increase in PEI led to precipitation. The ratio of 2:1 represents the best balance of cations and anions for gel formation.

**Figure 2:**
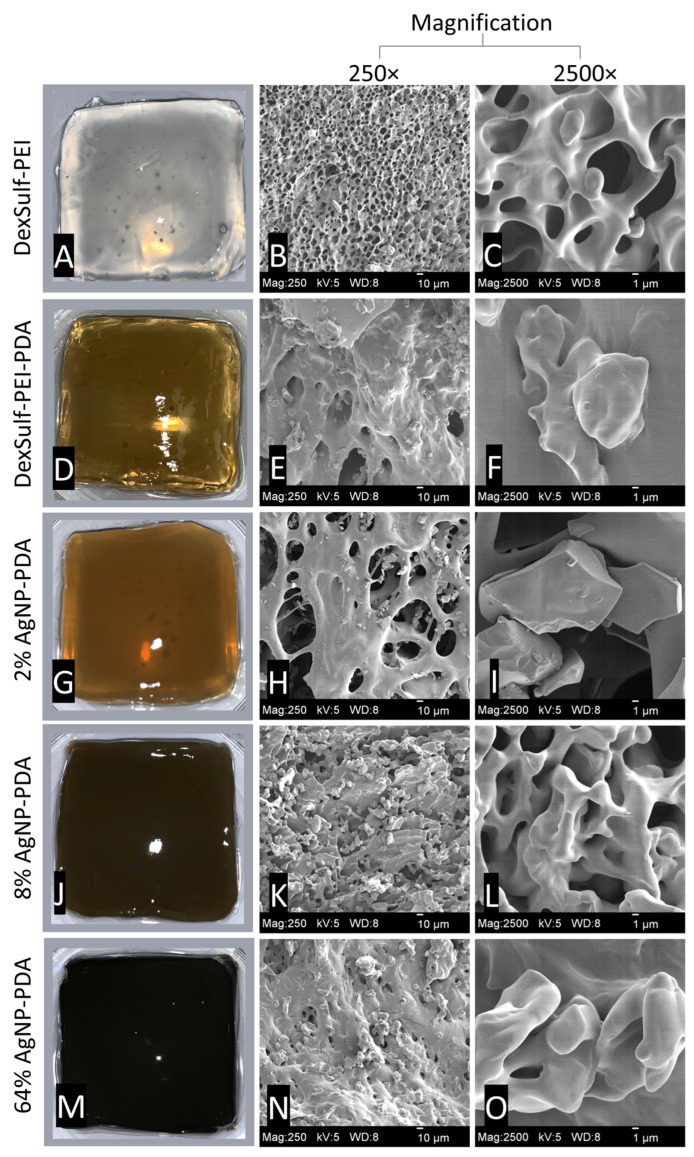
Representative images for hydrogels and SEM images at 250× and 2500× magnification: (**A**–**C**) DexSulf-PEI, (**D**–**F**) DexSulf-PEI-PDA, (**G**–**I**) 2% AgNP-PDA, (**J**–**L**) 8% AgNP-PDA and (**M**–**O**) 64% AgNP-PDA.

By substituting PEI with a polyethyleneimine-polydopamine copolymer, we produced hydrogels with darker hues, confirming the inclusion of polydopamine (Figure 2). This was achieved by maintaining a mass ratio of 2:1:0.17 for polyethyleneimine, dextran sulfate, and dopamine, respectively. PDA’s ability to act as a reducing agent for the synthesis of silver nanoparticles [19] was employed to generate silver nanoparticles in situ from varying concentrations of AgNO_3_ solutions (ranging from 0.01 to 0.32 M). The mass feed ratio of AgNO_3_ to dopamine ranged from 2% to 64%, resulting in increasingly darker and less transparent hydrogels, as shown in Figure 2. Hydrogels with varying concentrations of silver were prepared to identify the formulation with the lowest concentration of silver that still retained broad-spectrum antibacterial activity. In the reduction of AgNO_3_ to nanoparticles, the catechols of PDA are oxidized to quinones. Many of the attractive properties of PDA, such as antioxidant activity and biocompatibility, are attributed to its catechol functionality.

**Figure 3:**
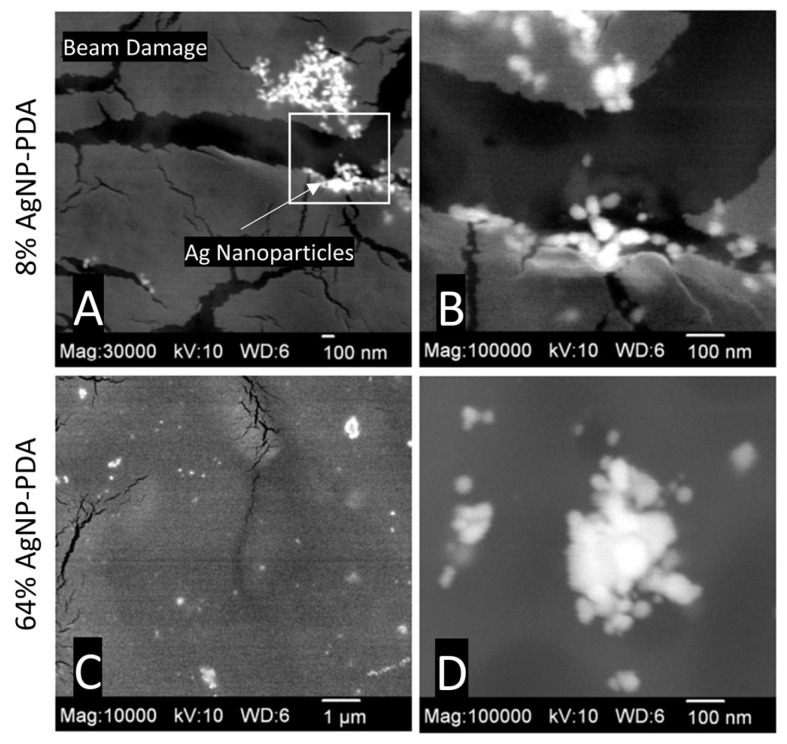
SEM images with BEI detector: (**A**,**B**) 8% AgNP-PDA at 30,000× (magnified region highlighted) and 100,000×, (**C**,**D**) 64% AgNP-PDA at 10,000× and 100,000×.

The DexSulf-PEI hydrogel exhibited a well-defined hierarchical pore structure, characterized by pores in the 10 µm size range (Figure 2B,C). However, the introduction of polydopamine in DexSulf-PEI-PDA led to a noticeable transformation, resulting in the loss of the porous structure (Figure 2E,F). Instead, we observed pores covered with a smooth material, accompanied by the presence of additional particles on the surface. When AgNO_3_ was incorporated into the formulation to create 2% AgNP-PDA hydrogels, the surface exhibited thin “platelets” (Figure 2H,I). As the concentration of AgNO_3_ was increased to 8%, we observed the emergence of smooth, rounded particles that obscured the underlying pore structure (Figure 2K,L). This trend persisted as the AgNO_3_ concentration was further increased, with the 64% AgNP-PDA hydrogel also displaying a similar covering of smooth particles. To confirm the presence of silver nanoparticles, SEM/EDX analysis was performed on 8% AgNP-PDA and 64% AgNP-PDA hydrogels, employing a backscattering electron detector (Figure 3). The EDX analysis revealed trace amounts of silver in the 8% AgNP-PDA hydrogel, while the signal was notably more pronounced, approximately eight times greater, in the 64% AgNP-PDA hydrogel (Figures S1 and S2). Further SEM examination indicated that the nanoparticles ranged in size from 25 nm to approximately 200 nm, with some silver nanoclusters extending up to about 800 nm.

**Figure 4:**
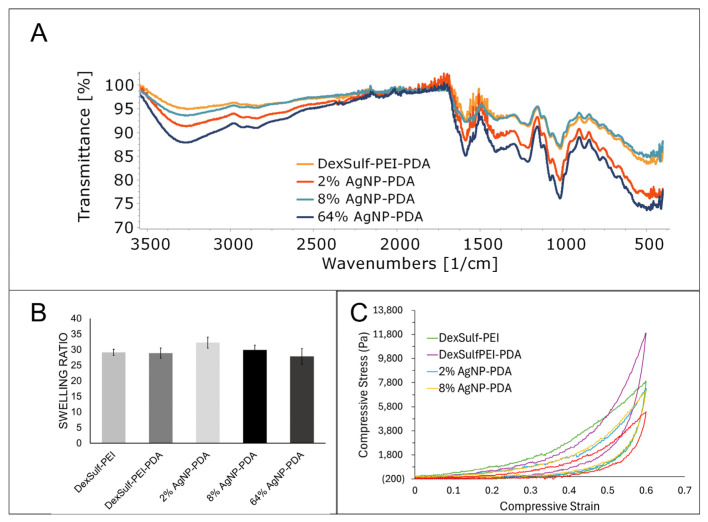
Characterization of DexSulf-PEI, DexSulf-PEI-PDA, 2%, 8% and 64% AgNP-PDA hydrogels: (**A**) FTIR spectra. (**B**) Swelling ratios in deionized water (*n* = 3, Mean ± SD). (**C**) Averaged compressive stress vs. compressive strain curves (*n* = 3).

No significant differences were found in the FTIR spectra as the major gel components dextran sulfate and PEI dominate the spectra (Figure 4A). The amount of dopamine used during gel synthesis is a fraction of what is used for PEI and dextran sulfate. Peaks attributed to PDA will be considerably smaller than the other starting materials and occur in similar regions. The characteristic OH and CH stretching vibrations for dextran sulfate were found at 3300, 2935 and 2850 cm^−1^. The amine bending vibration for PEI was identified at 1588 cm^−1^. The sulfate group’s presence is determined by the S=O asymmetric vibration at 1219 cm^−1^ [22]. 

The swelling ratio of a hydrogel measures the increase in volume as it absorbs water. It is a commonly used characterization method for hydrogels and provides information about the hydrophilicity and elasticity of gels. An analysis of hydrogel swelling ratios showed little variation between the formulations (Figure 4B). The DexSulf-PEI and PDA-containing hydrogel, DexSulf-PEI-PDA, showed near identical average swelling ratios of 29.1 and 28.9. A slight increase in the average swelling to 32.3 is observed in the 2% AgNP-PDA hydrogel when AgNO_3_ is added to the formulation. However, no consistent trend was observed with continued increase of AgNO_3_ as the swelling ratio lowers to 27.8 with the 64% AgNP-PDA hydrogel. This indicates that swelling is independent of PDA and silver nanoparticles and is primarily determined by the PEI and dextran sulfate ratio. Hydrogels formed by decreasing the PEI to dextran sulfate ratio from 2:1 to 1:1, were softer and displayed higher swelling ratios (Figure S3). For the systems examined, it is proposed that an increase in dextran sulfate shifts the balance of ionic groups, resulting in increased repulsion among the polymer chains and, consequently, greater swelling.

The mechanical characteristics of hydrogels were examined by compressive stress–strain curves and all hydrogels exhibited nonlinear hyperelastic behavior (Figure 4C). The stiffness was dependent on the hydrogel composition and showed a noticeable decrease with the inclusion of silver nanoparticles. To achieve a compressive strain of 60%, a compressive stress of 7915 Pa was required for the DexSulf-PEI hydrogel. The introduction of polydopamine led to a significant increase in the required compressive stress, approximately 1.5 times higher, at 11,904 Pa for the DexSulf-PEI-PDA hydrogel. This increase is likely due to the intermolecular interactions introduced by the addition of PDA. The addition of AgNO_3_ to the formulation resulted in a reduction in the compressive stress for a 60% strain, with hydrogels containing 2% and 8% AgNP-PDA showing nearly identical stress–strain curves and compressive stresses at 60% strain of 7296 Pa and 7177 Pa, respectively. The consumption of PDA to reduce silver would alter the PDA intermolecular interactions, and a substantial increase in silver content, with the 64% AgNP-PDA hydrogel, led to an even further reduction in compressive stress, down to 5393 Pa. 

### 2.2. Antibacterial Activity

**Figure 5:**
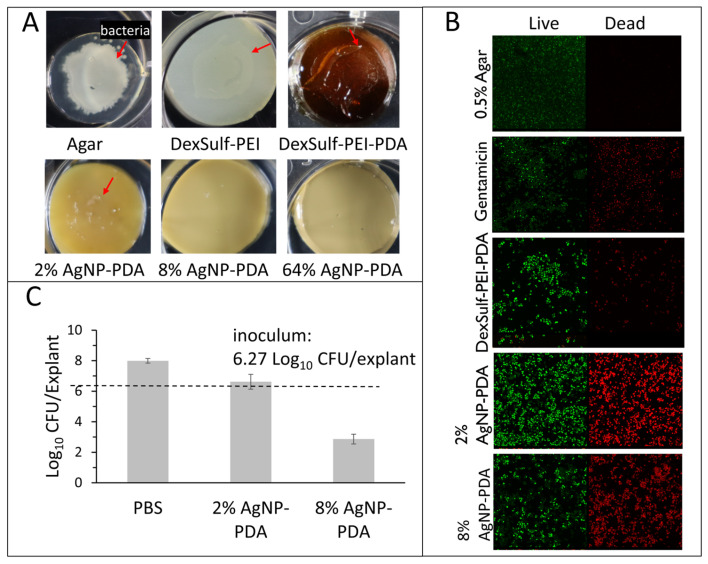
Antimicrobial studies with *S. aureus*. (**A**) DexSulf-PEI, DexSulf-PEI-PDA, 2%, 8% and 64% AgNP-PDA gels inoculated with 5 µL aliquots 10^8^ cells/mL of *S. aureus* and incubated for 24 h. Arrow indicates bacterial growth. (**B**) Bacterial live/dead assay of DexSulf-PEI-PDA, 2% AgNP-PDA and 8% AgNP-PDA hydrogels. Positive control was agar-treated with antibiotic gentamicin and negative control was untreated agar. (**C**) *S. aureus* infected human skin explant studies with PBS, 2% AgNP-PDA and 8% AgNP-PDA hydrogels. Unwounded human skin explants were inoculated for 24 h followed by 24-h treatment (*n* = 3, Mean ± SD).

Hydrogel antibacterial activity was confirmed against *P. aeruginosa*, *K. pneumoniae*, *E. coli* and *S. aureus*, which have been found to be the most common bacteria in multidrug-resistant septic wound infections [20]. To assess antibacterial activity, hydrogel surfaces were first inoculated with 5 μL bacterial aliquots with concentrations ranging from 10^2^ to 10^8^ cells/mL. Effective antibacterial efficacy was assessed as showing no visible bacterial growth or film on the hydrogel surface after 24 h incubation (Figure 5A). The positive control 0.5% agar, DeSulf-PEI and DexSulf-PEI-PDA all showed observable bacterial growth after 24 h incubation for *P. aeruginosa*, *K. pneumoniae*, *E. coli* and *S. aureus* at concentrations ranging from 10^2^ to 10^8^ cells/mL. The silver nanoparticle-containing hydrogels showed similar effectiveness against *S. aureus* and *E. coli* with 2% AgNP-PDA showing no observable bacterial growth for inoculation concentrations up to 10^6^ cells/mL. On 2% AgNP-PDA, *S. aureus* and *E. coli* growth was observed at the higher 10^8^ cells/mL inoculation concentration. Hydrogel formulations 8% and 64% AgNP-PDA both showed no observable *S. aureus* or *E. coli* growth at all bacterial concentrations examined. The 2%, 8% and 64% AgNP-PDA hydrogels were even more effective at inhibiting *P. aeruginosa* and *K. pneumoniae*, showing no observable growth over the full 10^2^–10^8^ cells/mL range examined. 

The successful inhibition of bacterial growth was further substantiated in the case of *S. aureus* through a bacterial live/dead assay (see Figure 5B). In this assay, propidium iodide (PI) serves as an intercalating red stain that is unable to penetrate healthy cells, making it effective for visualizing dead cells. On the other hand, SYTO 9 is a green, membrane-permeable intercalating stain used to visualize live cells. The live/dead staining of the control agar and the antibiotic gentamicin, displayed no bacterial cell death and substantial cell death, respectively. Unexpectedly the DexSulf-PEI-PDA formulation, lacking silver nanoparticles, exhibited some antibacterial activity, as evidenced by bacterial cell death. However, a significant enhancement in antibacterial activity was observed with the 2% and 8% AgNP-PDA formulations.

The efficacy of 2% and 8% AgNP-PDA formulations against *S. aureus* adherent to ex vivo human skin tissue was also investigated. The explants were inoculated with 6.27 CFU/explant and after 24 h treated with the hydrogel formulations for an additional 24 h. *S. aureus* concentrations after 24 h treatment for both 2% and 8% AgNP-PDA hydrogels were significantly lower than the phosphate-buffered saline (PBS) control (Figure 5C). The 2% AgNP-PDA hydrogel achieved a 1.23 Log reduction or 94.1% reduction from PBS treatment. The 8% AgNP-PDA hydrogel achieved a 5.07 Log reduction or >99.9% reduction from PBS treatment.

### 2.3. Biocompatibility and Wound Healing

**Figure 6:**
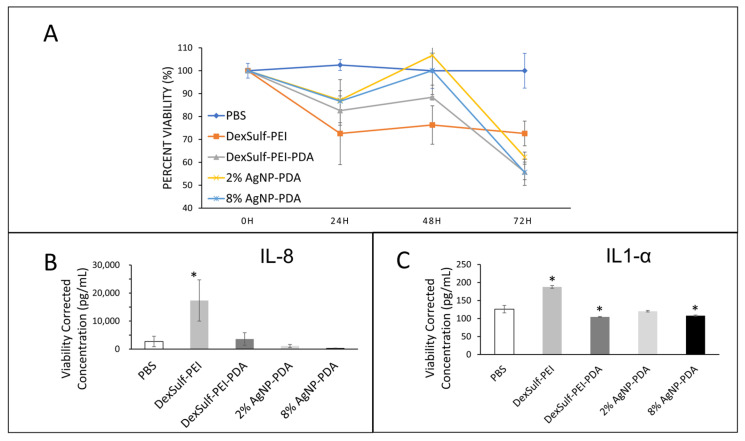
Biocompatibility studies on human skin explants with PBS (control), DexSulf-PEI, DexSulf-PEI-PDA, 2% AgNP-PDA and 8% AgNP-PDA hydrogels. (**A**) Cell viability over 72 h relative to PBS control. (**B**,**C**) ELISA after 24 h for interleukins IL-8 (left) and IL1-α (right). (*n* = 3, Mean ± SD). * indicates a significant difference from PBS treatment (*p*-value < 0.05).

The biocompatibilities of 2% and 8% AgNP-PDA hydrogels were examined by a cell viability assay over 72 h and compared to PBS as the control (Figure 6A). At the 24 h time point, DexSulf-PEI was the only formulation without polydopamine was the only hydrogel to decrease tissue viability below 75% compared to PBS control and was the only hydrogel significantly different from the control. At 48 h, the 2% AgNP-PDA and 8% AgNP-PDA formulations retained the most viability, showing no difference from the PBS control. At 72 h, all the hydrogel formulations examined became significantly different from PBS treatment with tissue viabilities decreased below 70% of the control. Over the course of the experiment, the gels were not replaced or rehydrated, and it is possible that the fall-off in viability at 72 h could be the result of the hydrogels drying.

Interleukins IL-8 and IL1-α are commonly used as biomarkers for inflammation and biocompatibility [21]. Examination of the pooled supernatant after 24 h of contact with human skin explants for IL-8 and IL1-α by ELISA revealed DexSulf-PEI as the only hydrogel in which the concentrations were significantly elevated compared to the control PBS treatment (Figure 6B,C). IL-8 was significantly greater (*p* = 0.0264) in the pooled supernatant of the DexSulf-PEI treatment group. IL1-α was also significantly greater (*p* = 0.0002) in the DexSulf-PEI treatment group. The increased concentration of IL-8 and IL1-α compared to control PBS indicates that the DexSulf-PEI hydrogel is irritating to skin and this mirrors our findings in cell viability (Figure 5A). The polydopamine-containing hydrogels DexSulf-PEI-PDA, 2% AgNP-PDA and 8% AgNP-PDA all showed comparable interleukin levels to control PBS. In fact, the DexSulf-PEI-PDA and 8% AgNP-PDA treatment groups had significantly lower IL1-α concentrations compared to PBS with *p* = 0.0238 and *p* = 0.0463, respectively. No correlations between biocompatibility and mechanical properties, such as swelling, and stiffness were observed. However, these findings indicate that PDA is necessary for biocompatibility. Dextran sulfate’s anionic properties make it attractive for various biomedical applications, including drug delivery, and as an anti-coagulant [23]. There are few records in the literature of dextran sulfate in wound healing applications [24]. In fact, it is widely used to induce inflammatory bowel disease-like colitis in animal models [25]. Our findings are that polydopamine ameliorates the inflammatory response to dextran sulfate. 

**Figure 7:**
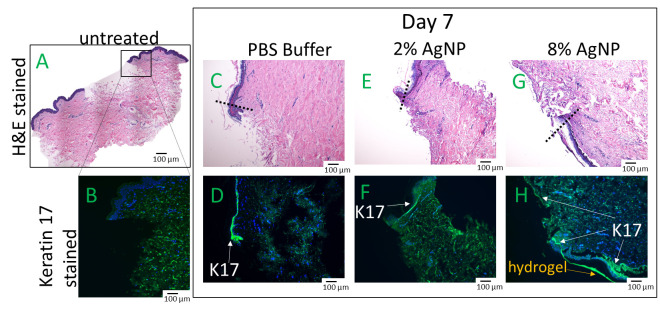
Hematoxylin & Eosin (H&E) and Keratin-17 (K17) stained human skin explants after 7 days of treatment with hydrogels. (**A**,**B**) Day 0 of untreated wounded skin. (**C**,**D**) PBS. (**E**,**F**) 2% AgNP-PDA. (**G**,**H**) 8% AgNP-PDA. Dashes indicate wound edge.

An ex vivo wound healing investigation using human explants was conducted to assess the impact of biocompatible formulations on the crucial wound healing process of re-epithelization. Wound closure relies on the migration of keratinocytes from the wound edge into the wound bed and cell proliferation occurs behind the migrating front [26]. Human skin explants were wounded and treated with formulations previously confirmed to exhibit biocompatibility, specifically DexSulf-PEI-PDA, 2% and 8% AgNP-PDA formulations. Histological examination with H&E staining revealed comparable levels of cell migration. Epidermal cells are observed forming a migration tongue at the wound’s leading edge, both in the gel-treated formulations and in the PBS control group (Figure 7C,E,G). However, a noteworthy distinction was observed in the 8% AgNP-PDA-treated tissue, where an extensively extended thin layer of keratinocytes covered the wound bed. This unique characteristic was further corroborated by Keratin-17 (K17) staining [27], which is upregulated in migrating and proliferating keratinocytes (Figure 7H). A significant accumulation of K17-stained cells was also observed behind the wound edge in response to the 8% AgNP-PDA formulation. These findings highlight the potential of this hydrogel formulation in promoting wound re-epithelization. Silver nanoparticles have been shown to be anti-angiogenic, inhibiting vascular endothelial growth factor-induced cell proliferation and cell migration [28]. These results demonstrate that PDA is likely able to counteract any anti-angiogenic properties that silver nanoparticles may present.

## 3. Conclusions

In summary, glucono-δ-lactone was demonstrated to be an effective acidifier for ionic cross-linking. The key role of PDA in promoting biocompatibility was highlighted, as evidenced by improved cell viability and a reduced inflammatory response, particularly in the presence of the known irritant, dextran sulfate. The reducing power of PDA was harnessed to create hydrogels with embedded silver nanoparticles, providing broad-spectrum antimicrobial activity. Furthermore, any anti-angiogenic properties of silver nanoparticles are likely mitigated by PDA. Ex vivo wound healing studies revealed that the 8% AgNP-PDA formulation exhibited substantial improvements in wound re-epithelialization, confirming their potential as wound healing materials.

## 4. Materials and Methods

### 4.1. Materials

#### 4.1.1. Materials

Dextran sulfate sodium salt (M_W_ ca > 500,000), dopamine hydrochloride and silver nitrate were obtained from Alpha Aesar (Tewksbury, MA, USA). 50% Polyethyleneimine (PEI, M_r_ 600,000–1,000,000) was obtained from Hampton Research (Aliso Viejo, CA, USA) and glucono-δ-lactone was obtained from EMD Millipore Corp (Burlington, MA, USA). For antibacterial studies, *E. coli* (ATCC 25922), *S. aureus* (ATCC 6538) *K. pneumoniae* (ATCC 13883), and *P. aeruginosa* (ATCC 27853) were used along with the LIVE/DEAD *Bac*Light Bacterial Viability Kit from Thermo Fisher Scientific (Waltham, MA, USA). 

#### 4.1.2. Characterization

Imaging of the Live/Dead assay was performed using a Leica TCS SP5 Confocal Laser Scanning Microscope (Wetzlar, Germany). Characterization with Attenuated Total Reflection (ATR)-FTIR was performed using a Nicolet iS10FTIR (Thermo Fisher Scientific, Waltham, MA, USA) and mechanical testing was performed with a Cellscale Univert (Waterloo, ON, Canada) uniaxial tension/compression tester. Field Emission Scanning Electron Microscopy (FESEM) was performed by Exel Laboratory Services (Dover, NJ, USA) with a JEOL 6300F cold cathode field emission scanning electron microscope (Tokyo, Japan). Imaging was performed at 5 kV beam voltage with a working distance of 6 mm. The vacuum conditions in the sample chamber were generally in the mid-10^−7^torr range. Energy dispersive X-ray analysis (EDX) was performed at 10 keV at a working distance of 39 mm. For nanoparticle analysis, a backscattered electron image (BEI) detector, which identifies the silver nanoparticles as bright spots in a darker background matrix, was employed. The lyophilized samples were coated with a thin film of Au/Pd prior to analysis. For nanoparticle analysis, the lyophilized samples were also vacuum dried at 100 °C for 2 h. The samples were coated with a thin film of Pt prior to SEM/EDX analysis. Biochemical assays were performed by Perfectus Biomed, LLC (Cheshire, UK). The statistical significance between means was determined by a one-way ANOVA followed by a Dunnett’s Test. Data is represented as a mean, with the error being a standard deviation. Probability values of *p* ≤ 0.05 are considered significant.

### 4.2. Hydrogel Synthesis

#### 4.2.1. DexSulf-PEI and DexSulf-PEI-PDA Hydrogel Synthesis

Dopamine HCl (25 mg) was added to a stirring solution of PEI (10% *w*/*v*, 3 mL) and mixed for 15 min. The formation of polydopamine was denoted by the darkening of the solution to dark brown. Dopamine was excluded from the procedure to produce DexSulf-PEI. The polyethyleneimine or polyethyleneimine-polydopamine copolymer solution was then added to a dextran sulfate (5% *w*/*v*, 3 mL) solution and stirred for 15 min. The pH was then lowered with the addition glucono-δ-lactone (0.9 g). The solution was stirred for 1 min and allowed to rest for 24 h, resulting in the formation of the polyelectrolyte hydrogel. 

#### 4.2.2. AgNP-PDA Hydrogel Synthesis

In order to prepare silver nanoparticle-containing hydrogels, 300 μL of AgNO_3_ solution of varying concentrations (0.01–0.32 M) was mixed with the dextran sulfate solution for 10 min prior to addition of the polyethyleneimine-polydopamine copolymer solution. The procedure was then followed as stated above.

### 4.3. Swelling Ratio

Each gel was partitioned into approximately four equal pieces and each piece was submerged in 50 mL of water for 24 h. After 24 h the swollen sections were removed, placed on paper towel and gently patted using Kimwipes to remove excess water. After weighing, the swollen gels were dried in a vacuum oven at 65 °C for 4 days to obtain their dried weight. The swelling ratio was calculated using the following equation: $\frac{w-w_{o}}{w_{o}}$, where *w* is the swollen weight and *w_o_* is the dried weight. The swelling ratio was determined by averaging 4 measurements and the error determined by their standard deviation.

### 4.4. Compression Studies

Circular hydrogel specimens for testing were prepared with diameters of 16 mm and a height of 6 mm. The specimens were preconditioned for 30 cycles at 10% strain with 5 s for compression and 5 s for recovery per cycle. Specimens were axially compressed to 60% strain with 5 s for compression and 5 s for recovery.

### 4.5. In Vitro Antibacterial Studies

#### 4.5.1. Hydrogel Surface Bacterial Inoculation

For a positive control 0.5% agar was used. Hydrogel formulations examined were DexSulf-PEI, DexSulf-PEI-PDA, 2% AgNP-PDA, 8% AgNP-PDA and 64% AgNP-PDA. The hydrogels were formed in petri dishes and 1.2 cm cutouts were transferred into 12-well cell culture plates, where they were UV sterilized on both sides for 5 min each. The gels were then soaked in 5 mL Miller Lysogeny broth for 48 h. Bacterial solutions with optical densities of 0.1 at 600 nm (>10^8^ cell/mL) were obtained and serial dilutions to 10^6^, 10^4^ and 10^2^ cells/mL. Excess media was removed from the hydrogels and then 5 μL of bacterial solutions was added to the surface of the media-soaked hydrogels and incubated at 37 °C for 24 h. Bacterial growth was confirmed visually.

#### 4.5.2. Bacterial Live/Dead Assay

For control experiments 0.5% agar and a 0.5% agar with Gentamicin (50 µg/mL) was used. The agar gel with Gentamicin (50 µg/mL), was formulated by adding 200 µL of a stock Gentamicin (100 mg/mL) solution to a petri dish, followed by the addition of 39.8 mL of 0.5% agarose solution. *S. aureus* (5 μL, 10^8^ cell/mL) was inoculated on gel surfaces and incubated for 24 h at 37 °C as described above. Following 24-h incubation, 20 µL of LIVE/DEAD *Bac*Light Viability Kit was added to the gel surface and incubated for 15 min in the dark at room temperature. This staining and incubation process was repeated for a second time. Agar, gentamicin, and DexSulf-PEI-PDA gels were transferred to a confocal dish and were imaged. For live/SYTO 9, excitation was 488 nm and emissions collected from 500 to 535 nm. For propidium iodide/dead, excitation was 543 nm and emissions collected from 604 to 644 nm. For the 2% and 8% AgNP-PDA gels, a thin layer from the gel surface was cut out from the site of cell inoculation and used for imaging due to the opaque and nontransparent nature of the gels.

### 4.6. Ex Vivo Human Skin Studies

An ex vivo human skin model was used to accomplish the studies described below and were performed by Perfectus Biomed, LLC. Deidentified human abdominal and eyelid skin discarded from elective plastic surgery procedures was obtained under IRB protocol #2018/12/2.

#### 4.6.1. Antibacterial Assay on Human Skin

Antibacterial activity was assessed for 2% and 8% AgNP-PDA using a colonized ex vivo human skin model. Full-thickness abdominal skin was placed on sterile saline gauze in a sealed container within 30 min post-surgery and transported to the laboratory. Single-species populations of *S. aureus* (ATCC 43300) were prepared to 5 × 10^8^ CFU/mL and colonized on 5 mm biopsied explants of ex vivo human skin. Following 24-h incubation at 37 °C, planktonic microorganisms were gently washed away with phosphate buffered saline (PBS) to leave only attached microorganisms. The hydrogel formulations were applied to cover the infected explants and incubated at 37 °C for an additional 24 h. Following the 24-h contact time, the explants were immersed in Dey-Engley neutralizing broth and the remaining viable bacteria was quantified using traditional microbiological plating. Tests were performed in dependent replicates of three. Log Reduction and percent reduction were calculated using the following equations:

$$Log\;Reduction=\log_{10}\frac{A}{B}$$

(1) $$Percent\;Reduction=100\left(\frac{A-B}{A}\right)$$

where *A* is the number of viable bacteria with PBS and *B* is the number of viable bacteria with hydrogel treatment.

#### 4.6.2. Human Skin Ex Vivo Biocompatibility Assays

Biocompatibility was examined by assaying inflammation biomarkers interleukins IL-8, IL1-α and cell viability. Intact human skin tissue explants (5 mm) from excised eyelids were obtained by a biopsy punch. Punched explants were transferred to the insert of a 6-well transwell cell culture plate and covered with hydrogels in triplicate, for an *n* = 3. The hydrogel-treated explants were incubated at 37 °C ± 2 °C and 5% CO_2_ with Roswell Park Memorial Institute (RPMI) 1640 medium for 24 h. Tissue viability was assessed via 3-[4,5-dimethylthiazol-2-yl]-2,5 diphenyl tetrazolium bromide (MTT) assay at 24 h, 48 h and 72 h. At the 24 h time point, 1 mL of pooled cell supernatants was collected and analyzed for interleukins IL1-α and IL-8 via an enzyme-linked immunosorbent assay (ELISA).

#### 4.6.3. Wound Healing Assay on Human Skin

Abdominal skin explants (5 mm) were wounded with a 2 mm partial thickness wound with a biopsy punch and transferred to sterile tissue culture trays containing RPMI 1640 media with 2% penicillin/streptomycin with the skin side up. Wounded explants were treated by placing hydrogels 2% and 8% AgNP-PDA within the wound bed. For a positive control experiment, 10 µL PBS was used. Explants were then incubated at 37 °C, 5% CO_2_ for 7 days. Every two days, 10 μL of PBS was added on top of the treatment area for hydration and the growth medium was replenished in the wells. At the collection timepoint, explants were transferred to 500 μL of formalin, the tissue processed, and hematoxylin and eosin (H&E) stained. Histology slides were visualized with an Olympus BX63 (Olympus LS, Tokyo, Japan) microscope at 63×. Re-epithelization was confirmed by staining with keratin-17 (K17) and 4′,6-diamidino-2-phenylindole (DAPI). Reserved unstained slides were heated in the microwave to melt paraffin, then deparaffinized in three changes of xylene for five minutes each. Sections were hydrated gradually through graded ethanol, with two changes of 100% ethanol for 15 min each and then two changes of 90% ethanol also for 15 min each. Slides were then dipped in three changes of deionized water to remove ethanol. Slides were heated in 10 mM sodium citrate buffer in the microwave until the buffer boiled. Microwave power was then reduced to 30%, and slides were heated for an additional seven minutes. Slides were allowed to cool, then washed in three changes of deionized water for two minutes each. Slides were incubated in blocking reagent for one hour (500 μL blocking reagent/slide), then washed in three changes of PBS for five minutes each. Slides were incubated with K17 antibody diluted to a concentration of 5 μg/mL in blocking reagent for 90 min. Excess reagent was removed, and the slides were washed in three changes of PBS for five minutes each. Slides were cover-slipped with aqueous mounting media with DAPI. Slides were imaged with fluorescence on the Olympus BX63 microscope.

## Figures and Tables

**Table 1:** Hydrogel formulations by mass ratio.

Hydrogels	Mass Ratio			
	Polyethylene Amine	Dextran Sulfate	Dopamine	AgNO_3_
DexSulf-PEI	2	1	—	—
DexSulf-PEI-PDA	2	1	0.17	—
2% AgNP-PDA	2	1	0.17	0.0034
8% AgNP-PDA	2	1	0.17	0.014
64% AgNP-PDA	2	1	0.17	0.108

## Data Availability

Data is contained within the article or Supplementary Materials.

## Supplementary Materials

The following supporting information can be downloaded at: https://www.mdpi.com/article/10.3390/gels10060363/s1, Figure S1: SEM/EDX analysis of 8% AgNP; Figure S2: SEM/EDX analysis of 64% AgNP; Figure S3: Swelling ratios in deionized water of hydrogels with 1:1 and 2:1 of PEI and Dextran Sulfate.

## References

[1] Liu Y., Ai K., Lu L. (2014). Polydopamine and Its Derivative Materials: Synthesis and Promising Applications in Energy, Environmental, and Biomedical Fields. Chem. Rev..

[2] El Yakhlifi S., Alfieri M.-L., Arntz Y., Eredia M., Ciesielski A., Samorì P., d’Ischia M., Ball V. (2021). Oxidant-dependent antioxidant activity of polydopamine films: The chemistry-morphology interplay. Colloids Surf. A Physicochem. Eng. Asp..

[3] Wang Q., Zhang R., Lu M., You G., Wang Y., Chen G., Zhao C., Wang Z., Song X., Wu Y. (2017). Bioinspired Polydopamine-Coated Hemoglobin as Potential Oxygen Carrier with Antioxidant Properties. Biomacromolecules.

[4] Zheng D., Huang C., Zhu X., Huang H., Xu C. (2021). Performance of Polydopamine Complex and Mechanisms in Wound Healing. Int. J. Mol. Sci..

[5] Fu Y., Yang L., Zhang J., Hu J., Duan G., Liu X., Li Y., Gu Z. (2021). Polydopamine antibacterial materials. Mater. Horiz..

[6] Jin Z., Yang L., Shi S., Wang T., Duan G., Liu X., Li Y. (2021). Flexible Polydopamine Bioelectronics. Adv. Funct. Mater..

[7] Siciliano G., Monteduro A.G., Turco A., Primiceri E., Rizzato S., Depalo N., Curri M.L., Maruccio G. (2022). Polydopamine-Coated Magnetic Iron Oxide Nanoparticles: From Design to Applications. Nanomaterials.

[8] Lynge M.E., van der Westen R., Postma A., Städler B. (2011). Polydopamine—A nature-inspired polymer coating for biomedical science. Nanoscale.

[9] Liebscher J., Mrówczyński R., Scheidt H.A., Filip C., Hădade N.D., Turcu R., Bende A., Beck S. (2013). Structure of Polydopamine: A Never-Ending Story?. Langmuir.

[10] O’Connor N.A., Syed A., Wong M., Hicks J., Nunez G., Jitianu A., Siler Z., Peterson M. (2020). Polydopamine Antioxidant Hydrogels for Wound Healing Applications. Gels.

[11] O’Connor N.A., Abugharbieh A., Yasmeen F., Buabeng E., Mathew S., Samaroo D., Cheng H.P. (2015). The crosslinking of polysaccharides with polyamines and dextran-polyallylamine antibacterial hydrogels. Int. J. Biol. Macromol..

[12] Kuo C.K., Ma P.X. (2001). Ionically crosslinked alginate hydrogels as scaffolds for tissue engineering: Part 1. Structure, gelation rate and mechanical properties. Biomaterials.

[13] Wang H., Deng H., Gao M., Zhang W. (2021). Self-Assembled Nanogels Based on Ionic Gelation of Natural Polysaccharides for Drug Delivery. Front. Bioeng. Biotechnol..

[14] Komoto D., Furuike T., Tamura H. (2019). Preparation of polyelectrolyte complex gel of sodium alginate with chitosan using basic solution of chitosan. Int. J. Biol. Macromol..

[15] Sun Y., Nan D., Jin H., Qu X. (2020). Recent advances of injectable hydrogels for drug delivery and tissue engineering applications. Polym. Test..

[16] Piras C.C., Smith D.K. (2020). Multicomponent polysaccharide alginate-based bioinks. J. Mater. Chem. B.

[17] Niu W., Liu X. (2022). Stretchable Ionic Conductors for Soft Electronics. Macromol. Rapid Commun..

[18] Zhao C., Zuo F., Liao Z., Qin Z., Du S., Zhao Z. (2015). Mussel-Inspired One-Pot Synthesis of a Fluorescent and Water-Soluble Polydopamine-Polyethyleneimine Copolymer. Macromol. Rapid Comm..

[19] Yang Z., Wu Y., Wang J., Cao B., Tang C.Y. (2016). In Situ Reduction of Silver by Polydopamine: A Novel Antimicrobial Modification of a Thin-Film Composite Polyamide Membrane. Environ. Sci. Technol..

[20] Pallavali R.R., Degati V.L., Lomada D., Reddy M.C., Durbaka V.R.P. (2017). Isolation and in vitro evaluation of bacteriophages against MDR-bacterial isolates from septic wound infections. PLoS ONE.

[21] Perkins M.A., Osterhues M.A., Farage M.A., Robinson M.K. (2001). A noninvasive method to assess skin irritation and compromised skin conditions using simple tape adsorption of molecular markers of inflammation. Skin Res. Technol..

[22] Cakic M., Nikolić G.M., Ilić L., Stanković S.M. (2005). Synthesis and FTIR characterization of some dextran sulphates. Chem. Ind. Chem. Eng. Q..

[23] Ramasundaram S., Saravanakumar G., Sobha S., Oh T.H. (2022). Dextran Sulfate Nanocarriers: Design, Strategies and Biomedical Applications. Int. J. Mol. Sci..

[24] Ammassam Veettil R., Marcano D.C., Yuan X., Zaheer M., Adumbumkulath A., Lee R., Isenhart L.C., Soriano N., Mhatre K., Joseph R. (2021). Dextran Sulfate Polymer Wafer Promotes Corneal Wound Healing. Pharmaceutics.

[25] Ohtsuka Y., Sanderson I.R. (2003). Dextran sulfate sodium-induced inflammation is enhanced by intestinal epithelial cell chemokine expression in mice. Pediatr. Res..

[26] Pastar I., Stojadinovic O., Yin N.C., Ramirez H., Nusbaum A.G., Sawaya A., Patel S.B., Khalid L., Isseroff R.R., Tomic-Canic M. (2014). Epithelialization in Wound Healing: A Comprehensive Review. Adv. Wound Care.

[27] Freedberg I.M., Tomic-Canic M., Komine M., Blumenberg M. (2001). Keratins and the keratinocyte activation cycle. J. Investig. Dermatol..

[28] Gurunathan S., Lee K.J., Kalishwaralal K., Sheikpranbabu S., Vaidyanathan R., Eom S.H. (2009). Antiangiogenic properties of silver nanoparticles. Biomaterials.

