# Supplementary material for: Antibacterial Silver Nanoparticle Containing Polydopamine Hydrogels That Enhance Re-Epithelization
Source: Gels. 2024 May 24;10(6):363. doi: 10.3390/gels10060363 (PMC11202472; doi:10.3390/gels10060363)
Supplement: Supplementary file 1 [file gels-10-00363-s001.zip › gels-2998810-supplementary.pdf]

Supplementary Information

Antibacterial Silver Nanoparticle Containing Polydopamine Hydrogels that Enhance Re-epithelization

Naphtali A. O'Connor[1,2,3,*], Abdulhaq Syed[1] , Ertan Kastrat[3] and Hai-Ping Cheng[3]

[1] Department of Chemistry, Lehman College of the City University of New York, Bronx, NY 10468 USA; abdulhaq.syed@lc.cuny.edu (A.S.)

[2] Ph.D. Program in Chemistry, The Graduate Center, City University of New York, New York, NY 10016 USA

[3] Ph.D. Program in Biochemistry, The Graduate Center, City University of New York, New York, NY 10016 USA; ertan.kastrat@lehman.cuny.edu (E.K); haiping.cheng@lehman.cuny.edu (H.C.)

* Correspondence: naphtali.oconnor@lehman.cuny.edu; Tel.: +718-960-8678

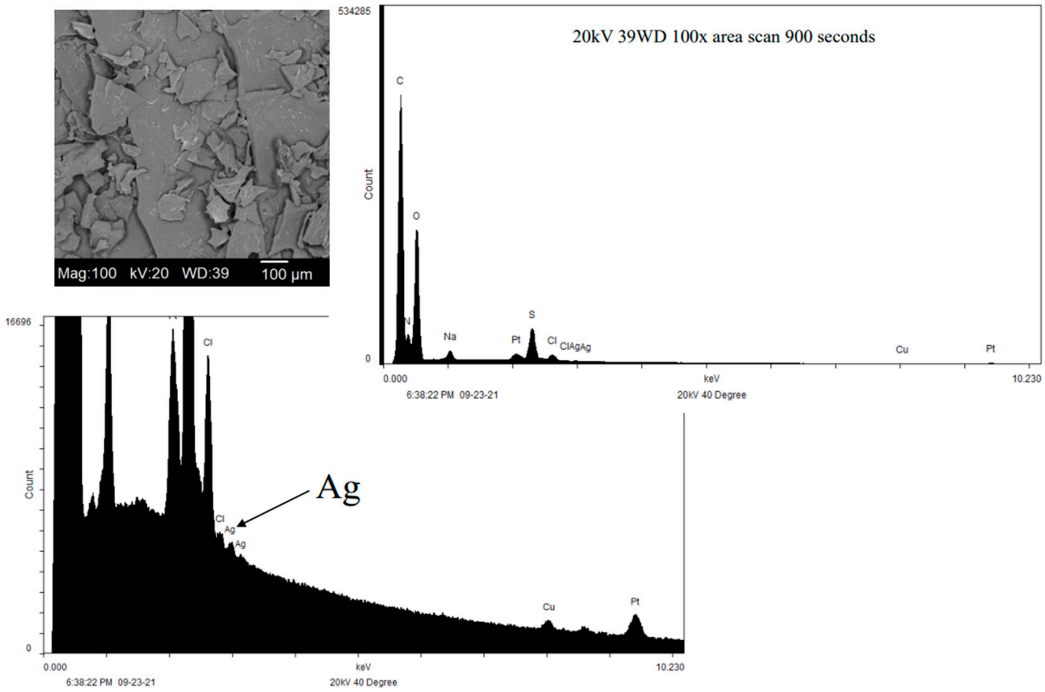

Figure S1. SEM/EDX analysis of 8% AgNP.

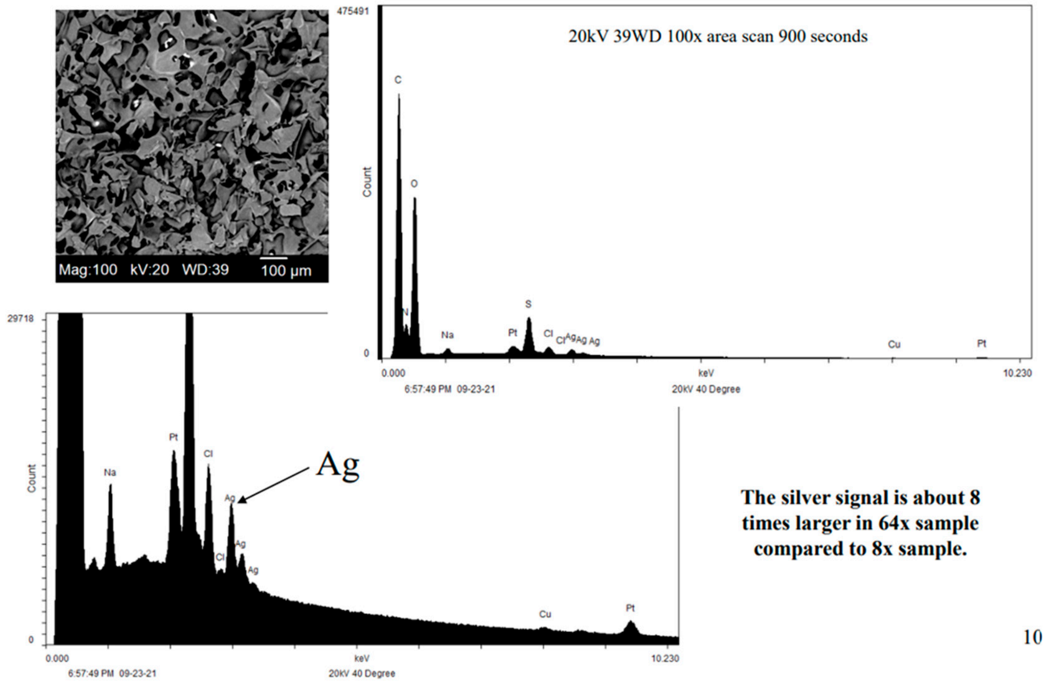

Figure S2. SEM/EDX analysis of 64% AgNP.

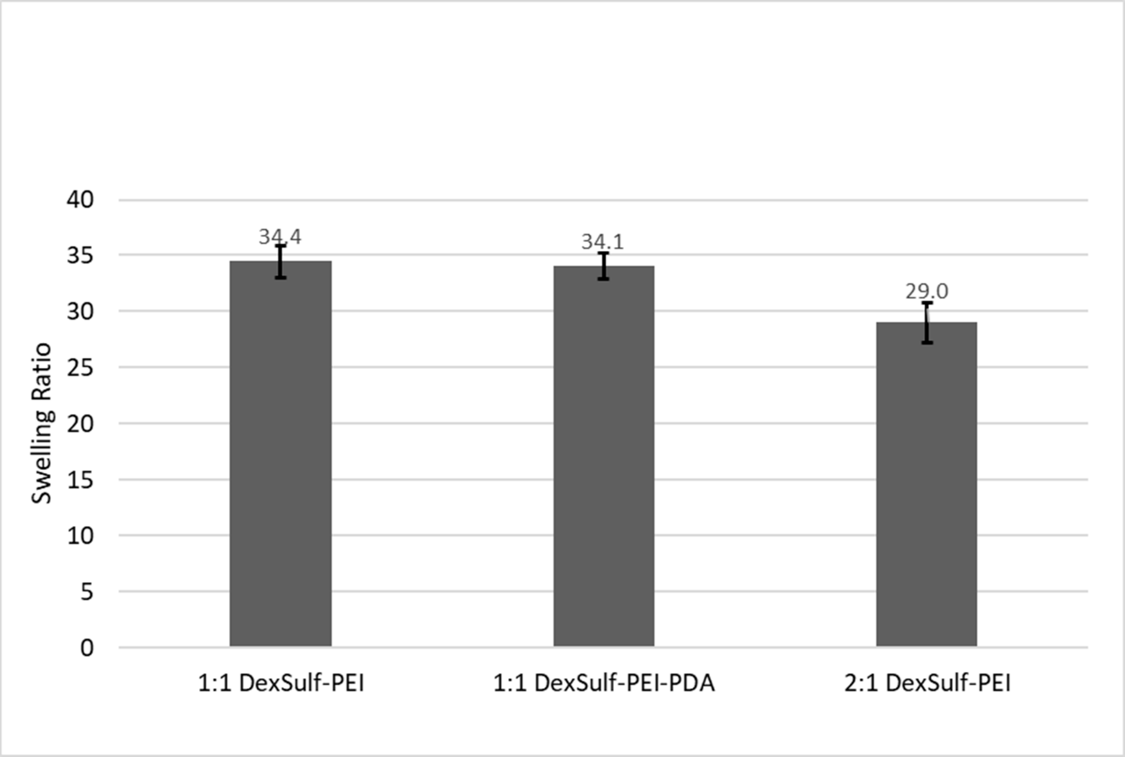

Figure S3. Swelling ratios in deionized water of hydrogels with 1:1 and 2:1 of PEI and Dextran Sulfate. (n=3, Mean ± SD).
